# Ambient temperature influences birds' decisions to eat toxic prey^☆^

**DOI:** 10.1016/j.anbehav.2013.07.007

**Published:** 2013-10

**Authors:** M. Chatelain, C.G. Halpin, C. Rowe

**Affiliations:** a Museum National d'Histoire Naturelle, Paris, France; b Centre for Behaviour and Evolution, Institute of Neuroscience, Newcastle University, Newcastle, U.K.

**Keywords:** aposematism, decision making, dietary cognition, mimicry, nutrient–toxin trade-off, state-dependent foraging

## Abstract

Aposematic prey warn predators of their toxicity using conspicuous signals. However, predators regularly include aposematic prey in their diets, particularly when they are in a poor energetic state and in need of nutrients. We investigated whether or not an environmental factor, ambient temperature, could change the energetic state of predators and lead to an increased intake of prey that they know to contain toxins. We found that European starlings, *Sturnus vulgaris*, increased their consumption of mealworm, *Tenebrio molitor*, prey containing quinine (a mild toxin) when the ambient temperature was reduced below their thermoneutral zone from 20 °C to 6 °C. The birds differed in their sensitivity to changes in ambient temperature, with heavier birds increasing the number of toxic prey they ate more rapidly with decreasing temperature compared to birds with lower body mass. This could have been the result of their requiring more nutrients at lower temperatures or being better able to detoxify quinine. Taken together, our results suggest that conspicuous coloration may be more costly at lower temperatures, and that aposematic prey may need to invest more in chemical defences as temperatures decline. Our study also provides novel insights into what factors affect birds' decisions to eat toxic prey, and demonstrates that selection pressures acting on prey defences can vary with changing temperature across days, seasons, climes, and potentially in response to climate change.

## Highlights

• We investigated the effect of temperature on birds' decisions to eat toxic prey.
• As it got cooler, birds were more likely to eat prey containing toxins.
• Heavier birds were more sensitive to changes in temperature.
• Selection pressures on prey defences will change over days, seasons and climes.

Many different types of forager are faced with foods that contain toxins as well as nutrients. For example, pollinators ingest toxic nectar from flowers (e.g. Adler 2000; Wright et al. 2013), herbivores graze on plants that contain various plant secondary metabolites (PSMs; e.g. Foley & Moore 2005; Marsh et al. 2006) and frugivores eat unripe fruits (e.g. Cipollini & Levey 1997). There are also numerous examples of predators including toxic aposematic prey in their diets (e.g. Fink & Brower 1981; Brower & Calvert 1985; Chai 1996; Pinheiro 1996; Speed et al. 2000). Aposematic prey advertise their toxicity to predators using conspicuous warning signals (Poulton 1890), an association that predators readily learn and subsequently they reduce their attacks on aposematically signalling prey (e.g. Gittleman & Harvey 1980; Roper & Redston 1987; Lindstrom et al. 1999; Halpin et al. 2008). But if naïve predators can learn to avoid aposematic prey based on their warning coloration, why do they still continue to eat them once they have been educated about their toxicity?

Educated predators' decisions, like those of other types of forager, are thought to reflect a trade-off between the costs of eating the toxin and the benefits of acquiring the nutrients (Sherratt 2003; Kokko et al. 2003; Sherratt et al. 2004). There is good evidence to support this idea, particularly from studies showing that predators eat more toxic prey when they have been food restricted (Swynnerton 1915; Sexton et al. 1966; Gelperin 1968; Williamson 1980; Chai 1996; Barnett et al. 2007, 2012), or when the number or size of alternative palatable prey in the environment is small (Lindström et al. 2004; Halpin et al. 2013). Under such conditions, the benefits to acquiring nutrients from defended prey will be increased relative to the costs of ingesting their toxins. Since a predator's energetic state influences its decision to eat toxic prey, extrinsic environmental factors could also affect predation of toxic prey. One key factor known to influence the energetic state and the decisions of predators when foraging on palatable prey is ambient temperature (e.g. Caraco et al. 1990; Bateson 2002). In this study, to understand better the selection pressures acting on insect defences, we investigated the impact of changing ambient temperature on the decisions of avian predators foraging on toxic insect prey.

Ambient temperature affects the costs of metabolism in animals (Schmidt-Nielsen 1990). All endotherms, including birds, have a thermoneutral zone (TNZ), which is a range of ambient temperatures at which the metabolic cost of maintaining body temperature is minimal: if the ambient temperature increases above or decreases below this range, the metabolic costs of thermoregulation increase (Brenner 1965; Lustick & Adams 1977; Biebach 1984; Dmi'el & Tel-Tzur 1985). Numerous studies have demonstrated that the basal metabolic rate (BMR) and energetic requirements of birds increase as the temperature decreases below their TNZ (e.g. Wunder et al. 1977; Broggi et al. 2004, 2007; Swanson 2010). One way that birds can meet the increasing metabolic costs of thermoregulation is to consume more palatable and more nutritious foods (e.g. Prince et al. 1965; Chaffee & Roberts 1971; Caraco et al. 1990), but they could also increase their ingestion of defended prey. Observations of wild birds foraging on monarch butterflies, *Danaus plexippus*, at overwintering sites in Mexico showed that the number of butterflies killed per day was higher on those days that were relatively cooler (Brower & Calvert 1985). Although this could be indicative of lower temperatures increasing the energetic requirements of birds, it could also be that there were fewer alternative prey available or that monarch butterflies were less active or easier to handle on cooler days. Therefore, there is as yet no direct evidence that ambient temperature affects predators' decisions to attack and eat defended prey.

In this experiment, we used climate-controlled chambers to investigate the effect of temperature on birds' decisions to attack prey that they know to contain quinine (a mild toxin). We used European starlings, *Sturnus vulgaris*, foraging on mealworm, *Tenebrio molitor*, larvae which has been established as a laboratory system to measure state-dependent decision making (Barnett et al. 2007, 2012; Skelhorn & Rowe 2007, 2010; Halpin et al. 2012). We predicted that a decrease in temperature would lead to an increased energetic requirement in the birds and that predation of toxic mealworms would also increase.

## Methods

### Subjects and Housing

A group of 50 European starlings were caught in the nonbreeding season (September/October 2011) in Northumberland under licence (Natural England 20113759) using a baited whoosh net. The birds (juveniles and adults) were immediately transported to the laboratory by car (a 30 min drive) and kept in two indoor aviaries (215 × 340 cm and 220 cm high) at Newcastle University. Each aviary had bark chippings on the floor, and ropes, boxes and branches for perches and cover. Birds were fed ad libitum poultry diet, mixed fruit and mealworms (mealworms were scattered into the bark chippings to promote natural foraging behaviour). Birds had constant access to drinking water and were provided with bathing water daily. The temperature of the aviary ranged between 15 °C and 18 °C, and was kept under a 14:10 h light cycle under high flicker rate (100 Hz) fluorescent lighting. Birds were individually marked using plastic colour rings, and were weighed each week and visually inspected daily by a trained technician during their time in captivity to ensure that they remained in good health.

Nine days before the start of the experiment, eight birds (four female and four male) were selected at random as experimental subjects and transferred to a climate-controlled chamber at 20 °C. Birds were individually housed in cages measuring 75 × 45 cm and 45 cm high with a flap facing the front through which prey could be presented. Although housed individually, birds were not visually or acoustically isolated from the other birds in the room, except during test sessions (see below). The cages were enriched with a bowl of water used for bathing, a litter tray containing wood chips for foraging and branches as perches. Birds were maintained on a 14:10 h light:dark photoperiod under high flicker rate (100 Hz) fluorescent lighting. Enriched vitamin water and a poultry maintenance diet were provided ad libitum except during experimental periods (see below). A piece of apple was also given at the end of each daily session. At the end of the experiment (April 2012), birds were returned to free-flight aviaries before being health checked by a vet, BTO ringed and released back to the wild at their site of capture. Although we were unable to monitor the birds following their release and subsequent dispersal, we have seen and recaught birds that we have previously released, demonstrating their ability to survive in the wild. The experiments were conducted under local ethical approval from Newcastle University (ERC Project ID No. 266).

### Preparation of Artificial Prey

Standard mealworms of a similar mass (19–21 mg) were injected with different solutions to make them either defended or undefended. Undefended prey were injected with 0.04 ml of water and defended prey were injected with 0.04 ml of 2% quinine sulphate solution (Quinine Sulfate, Sigma-Aldrich Q0132). Prey were injected with the same amount of solution to ensure that they were visually identical. The solutions were injected through the mouthparts of the mealworm using a hypodermic needle. Quinine is aversive to birds and is commonly used at low concentrations as a repellent in learning experiments with a range of different avian species (e.g. black-capped chickadees, *Parus atricapillus*: Alcock 1970; great tits, *Parus major*: Alatalo & Mappes 1996; European starlings: Skelhorn & Rowe 2006; domestic chickens, *Gallus gallus domesticus*: Halpin et al. 2008). Previous experiments have shown that quinine cannot be detected by olfaction or taste prior to ingestion when injected in this manner (Skelhorn & Rowe 2009, 2010).

### General Procedure

Birds were food deprived 1.5 h before the start of each session. Forty-five minutes before the start of a session, a sheet of opaque material was erected in front of the cages to prevent the test birds seeing conspecifics and the experimenter. The birds' behaviour was observed via video cameras connected to monitors. Each individual was given one session per day, during which a sequence of 16 single mealworms was presented. Each mealworm was presented in a petri dish through the flap on the front of the bird's cage. A single mealworm was presented in this manner every 3 min. The bird was given 1 min to eat it after which time the petri dish was removed. If the mealworm was attacked and eaten within 1 min, the petri dish was removed immediately. The experiment was divided into three consecutive phases: training (days 1–2), learning (days 3–9) and temperature manipulation (days 10–27).

### Training Phase

All birds were initially trained to eat unmanipulated mealworms that had not been injected with any solution. Birds received two sessions on consecutive days and the number of mealworms eaten was recorded. All birds were eating 15–16 mealworms per session by the end of day 2, and proceeded to the learning phase.

### Learning Phase

The birds received seven learning sessions, one per day for 7 consecutive days (days 3–9). In each session, eight undefended and eight defended prey were presented in a sequence in which two undefended and two defended prey were randomly delivered in every four presentations (this ensured that prey were equally distributed across the daily session). Undefended and defended prey were made visually distinct by placing a green or purple disc of paper under the mealworm in the petri dish. Colours were counterbalanced to control for any potential colour biases. Unique prey sequences were produced for each session for each bird to prevent birds using temporal cues to determine the palatability of prey. We recorded the numbers of undefended and defended prey eaten in each session. By the end of day 9, all birds had learned to discriminate between the two prey types (see Results) and the temperature manipulation phase began.

### Temperature Manipulation Phase

From day 10, birds continued to receive the same daily presentations of prey as in the learning sessions, but now we manipulated the temperature. We began the temperature manipulation phase with an additional day at 20 °C before decreasing the temperature from 20 °C to 6 °C by 2 °C every 2 days (15 days in total). We then increased the temperature to ensure that any change in behaviour was due to the change in temperature. We increased the temperature by 3 °C every day except for the last day, when the temperature was increased by 2 °C in order to reach the initial temperature of 20 °C (5 days in total; see Table 1 for details). Therefore, we ran different numbers of sessions at each temperature (one at 9 °C and 15 °C; two at 6 °C, 8 °C, 10 °C, 14 °C, 16 °C and 20 °C; and three at 12 °C and 18 °C). We chose this temperature range for two reasons: (1) it is below the TNZ of starlings and birds should be increasing their metabolic rate with decreasing temperature (Brenner 1965; Dmi'el & Tel-Tzur 1985); (2) it reflects the annual range of the mean daily maximum temperature at a local weather station, 25 km from Newcastle (http://www.metoffice.gov.uk/climate/uk/ne). Starlings have been reported as being kept between 12 °C and 28 °C in the laboratory (Asher & Bateson 2008), although we do not know what the impact of these different regimes is. Therefore, for welfare purposes (and following discussion with a Home Office Inspector), we monitored their daily intake of food and weighed the birds at each temperature below 12 °C. This would allow us to detect any sudden changes in the birds' energetic states and ability to cope with the decreasing temperature that would be a welfare concern.

### Measurements of Energetic State

Since we expected that a bird's energetic requirements would affect its intake of toxic prey, we took measures of their body condition (body mass, dietary intake and fat reserves) as well as their activity as temperature decreased from 20 °C to 6 °C. Birds were weighed at 0900 hours to reduce any effects of temporal fluctuations in body mass (Cresswell 1998; Broggi et al. 2004). We took two measurements at 20 °C, and single measurements at 10 °C, 8 °C and 6 °C (see Appendix for further details). We also recorded tarsus length and used this to calculate an index of body condition (body mass/tarsus length). We measured birds' dietary intake by weighing the amount of food (poultry diet and apple) that we gave to birds immediately following an experimental session and then reweighing the bowl and any remnants in the cage when we removed the bowl at the start of the food deprivation period for the following session on the next day. Since this was a noninvasive measure, we did this on a daily basis (see Appendix for further details). The subcutaneous fat reserves were estimated at 20 °C and at 6 °C using the BWG scale by an experienced ringer who was blind to the experimental manipulations (Redfern & Clark 2001). Fat reserves were scored between 1500 and 1600 hours to detect differences in mass gain during the day (see Appendix for further details). Finally, we measured activity by analysing 2 min of video taken randomly from eight sessions selected at 20 °C and 6 °C and counting the jumps from the perches to the ground that occurred during that period.

### Statistical Analyses

Statistical analyses were performed using SPSS v19.0 (SPSS Inc., Chicago, IL, U.S.A.). Where data did not conform to the assumptions of parametric statistics, we used nonparametric tests.

## Results

### Learning Phase

**Figure 1:**
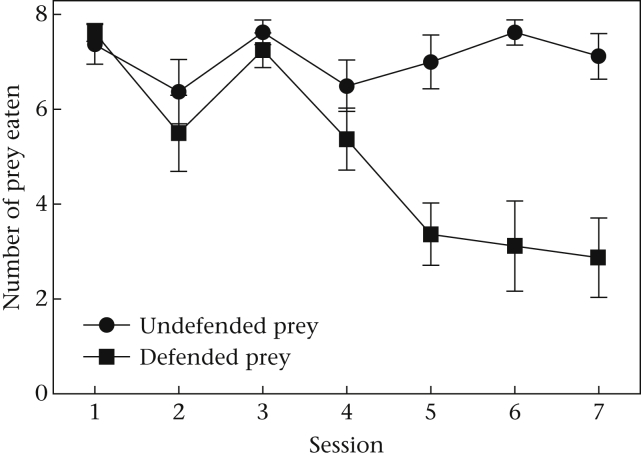
The mean ± SE numbers of defended and undefended prey eaten during the seven training sessions.

Birds learned to discriminate between defended and undefended prey during training: birds ate undefended and defended prey equally in the first session (Wilcoxon signed-ranks test: *Z* = 0.38, *N* = 8, *P* = 0.71), but ate significantly fewer defended than undefended prey in session 7 (*Z* = −2.34, *N* = 8, *P* = 0.02; Fig. 1). There was no significant difference in the numbers of defended prey eaten in sessions 5–7 (ANOVA: *F*_2,14_ = 0.10, *P* = 0.90). Taken together, these results demonstrate that the birds learned to discriminate between undefended and defended prey and were at asymptotic performance by the end of the learning phase (see Fig. 1). The number of defended prey that a bird ate at asymptote did not appear to be related to its body condition or need for nutrients. There was no significant relationship between the mean number of defended prey eaten at asymptote on days 5–7 and either body mass (Pearson correlation: *r* = −0.48, *P* = 0.23), body condition (*r* = −0.56, *P* = 0.15), fat score (*r* = −0.355, *P* = 0.39) or dietary intake (*r* = −0.25, *P* = 0.55).

### Temperature Manipulation Phase

**Figure 2:**
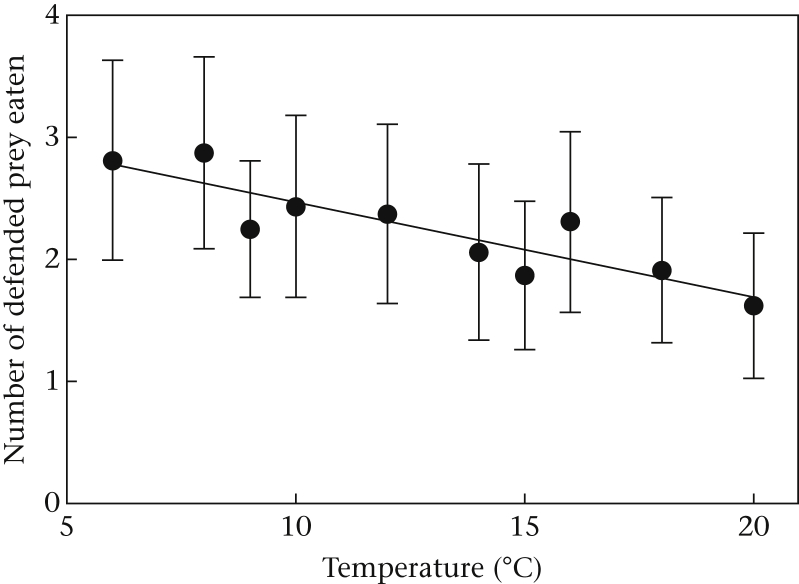
The mean ± SE number of defended prey eaten during sessions at each temperature during the temperature manipulation phase.

**Figure 3:**
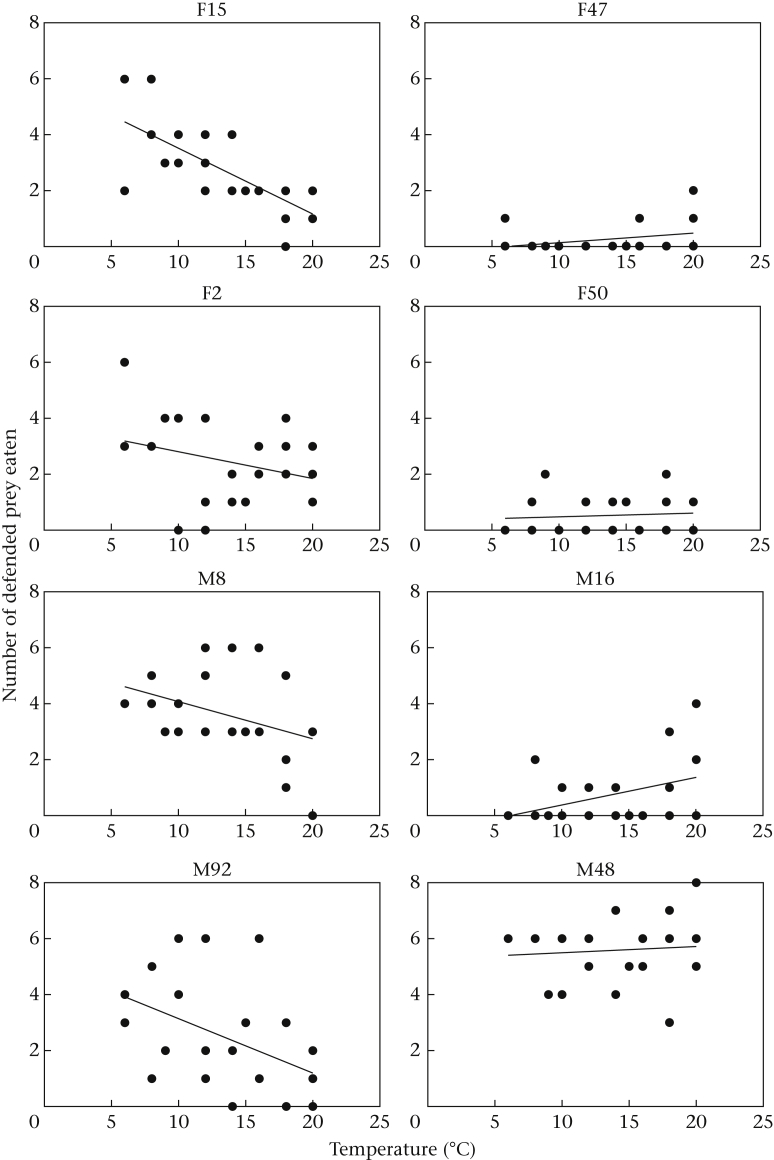
The linear regressions between temperature and the number of defended prey ingested for each individual bird. Each point is a data point for each day during the temperature manipulation phase, and the line is the linear regression through those points.

To investigate the effect of temperature on the ingestion of defended prey, we initially pooled the data from across the temperature manipulation phase and calculated the mean number of defended prey eaten at each temperature for each bird. We used this as our dependent variable and ran a general linear model with temperature as a covariate and bird as a random factor (Barnett et al. 2012). We found that as temperature decreased, the number of defended prey eaten increased (*F*_1,71_ = 12.76, *P* = 0.001; Fig. 2), although birds also differed in their responses to changing temperature (*F*_7,71_ = 45.21, *P* < 0.001; Fig. 3). The effect of temperature was also seen when we split the data set into a ‘temperature decreasing’ (days 8–21; see Table 1) and a ‘temperature increasing’ (days 22–27) phase (temperature decreasing: *F*_1,55_ = 7.99, *P* = 0.007; temperature increasing: *F*_1,39_ = 5.25, *P* = 0.027).

**Figure 4:**
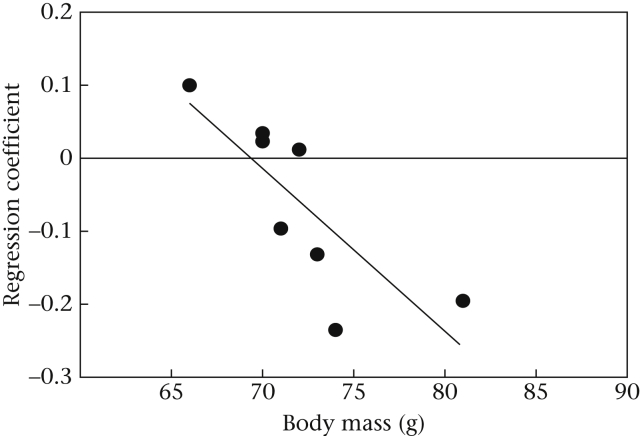
The relationship between birds' sensitivity to temperature (taken as the linear regression coefficient between temperature and the number of defended prey eaten) and body mass.

Since birds varied in their sensitivity to changing temperature, that is, the degree to which they altered their ingestion of defended prey in response to decreasing temperature (Fig. 3), we investigated whether predator energetic state could explain this variation. For each bird, we performed a linear regression between temperature and the number of defended prey eaten in each session to obtain a coefficient for the relationship for each bird (individual regression lines for each bird are shown in Fig. 3). We then correlated the regression coefficients with our initial measures of body condition for each bird taken at 20 °C prior to the start of the temperature manipulation phase. We found a significant negative correlation between the regression coefficient and body mass (Pearson correlation: *r* = −0.77, *P* = 0.03; Fig. 4), meaning that heavier birds increased their ingestion of defended prey more as temperature declined, that is, they were more sensitive to temperature change. We also found a near-significant negative correlation between temperature sensitivity and body condition (*r* = −0.69, *P* = 0.06), but no correlation with either fat score (*r* = −0.54, *P* = 0.17) or dietary intake (*r* = 0.26, *P* = 0.54). Taken together, these results suggest that initial body mass (and possibly also condition) affected the degree to which birds increased their intake of defended prey in response to changes in temperature. This could have been the result of heavier birds carrying less fat reserves or needing to eat more food for basic metabolic processes. However, we found no relationship between initial body mass and either dietary intake (*r* = 0.03, *P* = 0.71) or fat scores (*r* = 0.11, *P* = 0.43) at 20 °C before the temperature was reduced.

**Figure 5:**
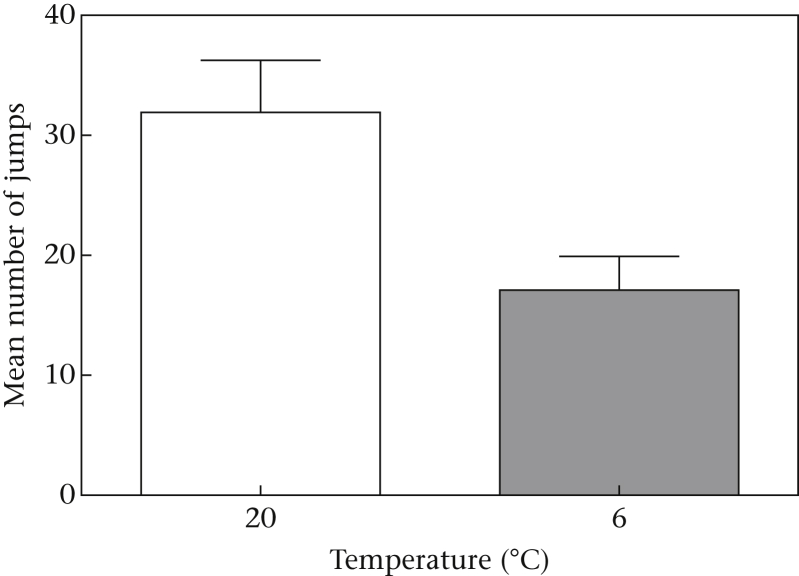
The mean ± SE number of jumps in the cage reflecting the activity of the birds at 20 °C and 6 °C.

We also investigated whether or not our measures of state differed between 20 °C and 6 °C (see Appendix for full details of the data collected and used for these analyses). We found no effect of temperature on the birds' masses (mean ± SE = 72.1 ± 1.53 g at 20 °C and 72.6 ± 1.24 g at 6 °C; paired *t* test: *t*_7_ = −0.0.88, *P* = 0.41), fat scores (mean ± SE = 2.38 ± 0.18 at 20 °C and 2.31 ± 0.16 at 6 °C; Wilcoxon signed-ranks test: *Z* = −0.58, *N* = 8, *P* = 0.56) or dietary intake (mean ± SE = 37.7 ± 1.44 g at 20 °C and 34.9 ± 1.86 g at 6 °C; paired *t* test: *t*_7_ = 1.75, *P* = 0.12). However, we did find that birds jumped in their cages less and were less active at 6 °C compared to 20 °C (independent samples *t* test: *t*_14_ = 2.85, *P* = 0.01; Fig. 5). Therefore, lowering the temperature appeared to reduce birds' movement rather than increasing their dietary intake, mass or fat stores.

## Discussion

Our study clearly demonstrates that reducing the ambient temperature can increase the predation of toxic prey by avian predators. This is the first time that an abiotic environmental factor has been shown to affect predatory decisions on signalling prey known to contain toxins. While previous experiments have shown that birds increase their overall dietary intake, particularly of energy-rich foods, at lower temperatures (Prince et al. 1965; Chaffee & Roberts 1971; Sprenkle & Blem 1984; Wansik & Tinbergen 1994), this is the first demonstration that birds eat more toxic prey as temperature decreases. This is most likely to be in response to an increased need for energy at lower temperatures, which increases the benefits of eating the nutrients relative to ingesting the toxins (see also Barnett et al. 2007, 2012). Our results suggest that selection pressures on insect defences could vary with temperature across days, seasons or environmental climes, such as latitude and elevation. Temperature-dependent predation on toxic prey is predicted to change the costs and benefits to warning coloration and investment in defences in prey.

First, decreasing temperature will increase the costs of being conspicuous for aposematic prey. Conspicuous coloration already carries a significant detectability cost, although this can potentially be offset by predators learning to associate conspicuous coloration with toxicity more quickly with conspicuous compared to cryptic signals (e.g. Gittleman & Harvey 1980; Alatalo & Mappes 1996; Lindström et al. 1999). Our results suggest that if predators more readily ingest toxic prey at low temperatures, warning coloration will become even more costly as temperature decreases. This could lead to selection for less conspicuous warning signals in colder environments, for example, through the evolution of patterns that make prey cryptic at a distance (Tullberg et al. 2005) or by a reduction in the size or coloration of the colour signal. While increasing latitude tends to be associated with smaller or duller signals in aposematic prey, this is generally ascribed to the thermoregulatory benefits of increasing the amount of melanin in the signal (Lindstedt et al. 2009; Mochida 2011; Hegna et al. 2013). However, detectability costs could also be important in selection for less colourful signals, particularly for prey for which the costs of predation increase more rapidly than the benefits of thermoregulation with decreasing temperature (Hegna et al. 2013).

Second, increased predation of toxic prey at lower temperatures could also select for increased toxicity of aposematic prey. This is likely to evolve only if the benefits from the associated reduction in predation outweigh any costs of increased toxicity, for example, reduced growth rates or lower fecundity (Pasteels et al. 1983; Cohen 1985; Rowell-Rahier & Pasteels 1986; Zalucki et al. 2001). However, there appear to be some insect species for which the costs of possessing toxins appear minimal (Kearsley & Whitham 1992), suggesting that increased toxicity could be selected for in at least some cases. An alternative strategy would be to aggregate in colder weather in order to pool defences to saturate a predator's detoxification pathways. Some aposematic insects, such as seven-spot ladybirds, *Coccinella septempunctata*, and monarch butterflies, often aggregate in winter (Brower & Calvert 1985; Holloway et al. 1991), a strategy that is known to enhance individual survival (Alatalo & Mappes 1996; Riipi et al. 2001; Finkbeiner et al. 2012). Perhaps this strategy is particularly effective at lower temperatures when predators are more willing to ingest toxic prey, since it ensures that a predator's toxin burden is reached, allowing the majority of individuals to survive.

Finally, our results also suggest that the costs and benefits of mimicry may change with temperature. For example, in Batesian or quasi-Batesian mimicry, where a visual mimic has a parasitic relationship with a more toxic model (Bates 1862; Speed 1993), the benefits to the mimic may rapidly decrease with decreasing temperature. This is because the benefits of looking the same as a toxic model will be much reduced and predators will be less likely to reject a model or a mimic upon encounter. If Batesian model–mimic complexes become more acceptable as food at lower temperatures, the cost of the mimic on the model may also increase at lower temperatures. To our knowledge, there has been no investigation of how the costs and benefits to mimicry might change with temperature, but it provides an interesting avenue for future research, both in the laboratory and in the field.

Although we found a main effect of temperature on the predation of toxic prey, our birds varied in their sensitivity to decreasing temperature. We found that heavier birds (and/or possibly those in better body condition) increased their ingestion of defended prey more rapidly than lighter birds. One possible explanation for this is that heavier individuals had a higher BMR and that their need for nutrients was higher than that of individuals with lower body mass (Selman et al. 2001; Speakman et al. 2004; Boratyński & Koteja 2009). However, heavier individuals did not have higher dietary intake than lighter individuals. Perhaps instead, these birds were more sensitive to the food deprivation period prior to the start of experimental sessions (possibly owing to a higher BMR), and that this increased their motivation to acquire nutrients. Although it seems likely that foraging decisions were based upon changes in the energetic requirements of birds, we cannot rule out the possibility that the heavier birds were somehow better able to process toxins at lower temperatures compared to lighter birds, although the possible mechanisms by which this would occur are not clear. Given that our study was purely behavioural, we are unable to discriminate between these different possibilities at this stage.

Although we found an effect of body mass on the birds' sensitivity to ingest toxic prey with decreasing temperature, we found no effect of either body mass, condition, dietary intake or fat scores on the number of defended prey eaten by an individual when at asymptote during the learning phase the learning phase. This is perhaps surprising given that we would expect energetic state to be important in an individual's decision to eat toxic prey (Barnett et al. 2007, 2012). Although the sample size was small in this experiment, we have been unable to find an effect of body mass or condition on the number of defended prey eaten at asymptote in a larger sample of birds used in a previous experiment (*N* = 33 birds; C. G. Halpin & C. Rowe, unpublished data, from Halpin et al. 2012). There are two possible reasons for this. First, our measures do not reliably measure energetic state, and other factors that we did not measure could be affecting nutrient requirements, for example, age, health or gut microflora (see Murphy 1996 for full list). Second, it could be that factors other than energetic state are also involved in determining birds' decisions to forage on toxic prey. Perhaps the most obvious is birds' abilities to detoxify ingested toxins. Brower's work on blue jays, *Cyanocitta cristata*, showed that individual birds within a population varied in their ability to cope with ingested cardenolides from monarch butterflies (Brower et al. 1968; Brower & Glazier 1975). It is therefore entirely possible that our starlings also differed in their abilities to detoxify quinine, because of either genetic variation or exposure to toxins prior to capture. Clearly, we are only just beginning to understand the mechanisms underlying birds' decisions to eat toxic prey, and future experiments could build on our findings to explore the interaction between genetic, physiological and cognitive factors in how predators decide to forage on toxic prey.

While our experiment has focused on the predation of toxic prey by avian predators, two studies have explored how temperature affects mammalian herbivores grazing on plants containing PSMs. In contrast to our own findings, neither of these studies found that herbivores increased their ingestion of toxic plant material when the ambient temperature was reduced below their TNZ (Stapley et al. 2000; Dearing et al. 2008). Because of the paucity of studies investigating how ambient temperature affects animals foraging on toxic foods, it is hard to know whether the differences between our study on avian predators and those on mammalian herbivores are due to comparing across taxa, foraging modes or different laboratory protocols. Clearly, a better understanding of the relationship between temperature, toxicity and energetics is required if we are to understand selection pressures acting on toxic animals and plants (see also Dearing et al. 2008).

Of course, any study investigating how foraging decisions change with temperature is potentially relevant for understanding how climate change may affect predator–prey dynamics and animal populations. While our study is perhaps limited in that we only manipulated temperature over several weeks, it does suggest that long-term changes in temperature may also affect selection pressures acting on aposematic prey and their mimics. Our results suggest that ambient temperature affects the evolutionary dynamics of prey defences, and raises interesting future avenues for research in aposematism and mimicry.

## Figures and Tables

**Figure A1:**
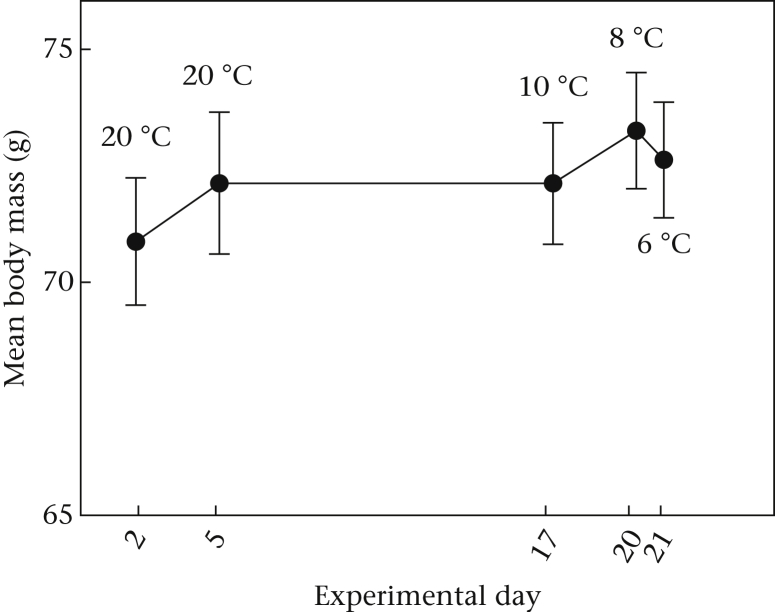
The mean ± SE mass of the birds taken as the temperature was reduced from 20 °C to 6 °C.

**Figure A2:**
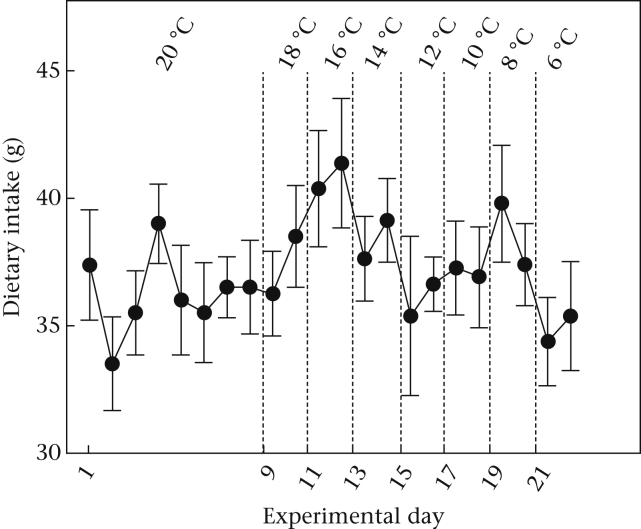
The mean ± SE daily dietary intake of birds on experimental days 1–22. Vertical lines denote a change in temperature.

**Figure A3:**
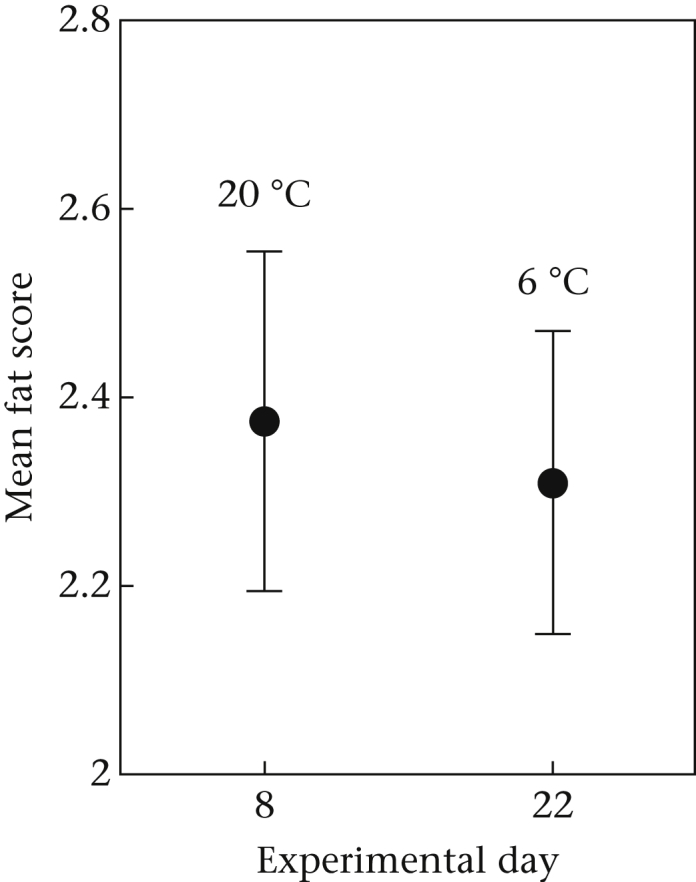
The mean ± SE fat score of the birds at 20 °C (day 8) and 6 °C (day 22).

**Table 1:** The temperatures used across the 20 days of the temperature manipulation phase (day 8–27)

Day	Temperature (°C)
8	20
9–10	18
11–12	16
13–14	14
15–16	12
17–18	10
19–20	8
21–22	6
23	9
24	12
25	15
26	18
27	20

## Appendix: Body condition measurements

The time points during the experiment at which we collected data on body mass, dietary intake and fat stores differed. For consistency, we compared data collected at 20 °C and 6 °C; however, here we report the full data collection methods and the data collected at all sampling points for each of our three measures.

### Body Mass

Since measuring the birds' masses could disrupt their behaviour, we took measures during the learning phase at 20 °C (days 2 and 5), and three in the temperature manipulation phase on experimental days 17 (10 °C), 20 (8 °C) and 21 (6 °C). These latter measurements also ensured that our birds were not rapidly losing weight in response to decreasing temperature. The data from all days are presented in Fig. A1. To compare whether birds' masses differed between 20 °C and 6 °C, we compared the data between day 5 (the last measure at 20 °C prior to the temperature manipulation) and day 21 (the only measure at 6 °C).

### Dietary Intake

We took daily measures of the total mass of food eaten by birds during the learning phase at 20 °C and the temperature manipulation phase as the temperature was reduced from 20 °C to 6 °C (days 1–22). The data from all days are presented in Fig. A2. To compare whether birds' intake differed between 20 °C and 6 °C, we compared the data between day 8 (the last measure at 20 °C prior to the temperature manipulation) and day 22 (the last measure at 6 °C).

### Fat Scores

To maximize the opportunity for detecting changes in fat storage, we fat scored the birds on day 8 (20 °C) and day 22 (6 °C) which reduced handling and potential stress to the birds. The data are presented in Fig. A3.
